# Adding Pay-for-Performance Program to Routine Care Was Related to a Lower Risk of Depression Among Type 2 Diabetes Patients in Taiwan

**DOI:** 10.3389/fpubh.2021.650452

**Published:** 2021-10-13

**Authors:** Wei-Cheng Lian, Hanoch Livneh, Hui-Ju Huang, Ming-Chi Lu, How-Ran Guo, Tzung-Yi Tsai

**Affiliations:** ^1^Division of Metabolism and Endocrinology, Department of Internal Medicine, Dalin Tzuchi Hospital, The Buddhist Tzuchi Medical Foundation, Chiayi, Taiwan; ^2^School of Medicine, Tzu Chi University, Hualien, Taiwan; ^3^Rehabilitation Counseling Program, Portland State University, Portland, OR, United States; ^4^Department of Nursing, Dalin Tzuchi Hospital, The Buddhist Tzuchi Medical Foundation, Chiayi, Taiwan; ^5^Division of Allergy, Immunology and Rheumatology, Dalin Tzuchi Hospital, The Buddhist Tzuchi Medical Foundation, Chiayi, Taiwan; ^6^Department of Environmental and Occupational Health, College of Medicine, National Cheng Kung University, Tainan, Taiwan; ^7^Department of Occupational and Environmental Medicine, National Cheng Kung University Hospital, Tainan, Taiwan; ^8^Occupational Safety, Health, and Medicine Research Center, National Cheng Kung University, Tainan, Taiwan; ^9^Department of Medical Research, Dalin Tzuchi Hospital, The Buddhist Tzuchi Medical Foundation, Chiayi, Taiwan; ^10^Department of Nursing, Tzu Chi University of Science and Technology, Hualien, Taiwan

**Keywords:** type 2 diabetes, pay-for-performance, depression, cohort study, risk

## Abstract

**Background:** Patients with type 2 diabetes (T2DM) often experience depression during treatment, negatively influencing their treatment compliance and clinical outcomes. Recently, the pay-for-performance (P4P) program for chronic diseases, with high-cost and high-risk feature, such as T2DM, has been implemented and has been operational for several years. Nevertheless, its effect on the risk of developing depression among T2DM cases is unknown. This study aims to explore the association of P4P use with the subsequent risk of developing depression among these patients.

**Methods:** This cohort study used a nationwide health insurance database to identify patients 20–70 years of age newly diagnosed with T2DM who enrolled in the P4P program between 2001 and 2010. From this group, we enrolled 17,022 P4P users and then 17,022 non-P4P users who were randomly selected using propensity-score–matching. Enrolled patients were followed until the end of 2012 to record the occurrence of depression. The Cox proportional hazards regression was utilized to obtain the adjusted hazard ratio (aHR) for P4P use.

**Results:** During the study period, a total of 588 P4P users and 1,075 non-P4P users developed depression at incidence rates of 5.89 and 8.41 per 1,000 person-years, respectively. P4P users had a lower depression risk than did non-P4P users (aHR, 0.73; 95% Confidence Interval, 0.65–0.80). This positive effect was particularly prominent in those receiving high-intensity use of the P4P program.

**Conclusion:** Integrating P4P into routine care for patients with T2DM may have beneficial effects on curtailing the subsequent risk of depression.

## Introduction

Depression is a chronic mental disorder that impacts cognitions, moods, behaviors, and physical well-being, thereby affecting quality of life (1). Depression affects approximately 350 million people worldwide and is particularly common among individuals with chronic diseases (2). For example, the association between depression and type 2 diabetes (T2DM) has been recognized for many years. One epidemiological study suggested that at least one-third of people with diabetes suffer from clinically relevant depressive disorders (3). A meta-analysis of 11 studies reported a 24% increased risk of incident depression in people with T2DM, compared to those without T2DM (4). Notably, the combination of diabetes and depression poses critical clinical challenges to the healthcare system. T2DM patients suffering from concomitant depression have an estimated 52% increase ($10,016 vs. $15,155) in annual medical expenses over those without depression (5), and more than double the likelihood of mortality due to poor glucose regulation and poor compliance with diabetes treatments (6). Therefore, an early implementation of effective diabetes management is important to prevent or lessen the susceptibility to depression among such patients (7).

The present standard modality for decreasing depression onset among diabetic patients is the use of anti-depressant medications. Some of these medications, however, may induce adverse side effects such as weight gain and decreased glycemic control (4, 8, 9). In response to this, another option, the pay-for-performance (P4P) program for patients with high-cost and high-risk chronic diseases, has raised attention due to its feature (10). The aim of P4P programs is to establish a complete “patient-centered” co-care model for self-care related to health education, consultation, referral system establishment, and resource linking. Moreover, in order for the patient to stabilize the disease and improve quality of life, it is necessary to actively participate in programs, and regularly follow-up with case management, allowing patients to receive regular visits and evaluate physician-prescribed medications to reduce the development of any complications (11). A growing body of scientific evidence indicates that implementing P4P significantly increases the quality and satisfaction of medical care for diabetic patients (12), and diminishes the burden of disease-generated complications (13, 14). At present, no data are available on the success of P4P in decreasing the risk of depression among T2DM patients. To address this concern, we used a national insurance database to compare depression risks between patients with T2DM who received and who did not participate in the P4P program.

## Methods

### Data Source

The Longitudinal Health Insurance Database (LHID) was utilized as the data source in this retrospective cohort study. The LHID is a sub-dataset of the National Health Insurance Research Database (NHIRD) of Taiwan, made up of one million randomly sampled people with over 10 years of available follow-up data. It has been established that these recruited individuals have similar age and sex distributions to the general population of Taiwan because of a multistage stratified systematic sampling method performed by the Bureau of National Health Insurance (NHI) to ensure the representativeness of the sampling (15). This database has been previously used in numerous research reports (16, 17). This database compiled (i) demographic information of enrollees; (ii) health insurance claims data; (iii) diagnostic codes; (iv) contracted pharmacies; and (v) medical examination information on persons under the NHI program. This study was conducted in accordance with the Helsinki Declaration, and was approved by the local institutional review board and ethics committee of Buddhist Dalin Tzu Chi Hospital (No. B10004021-3).

### Study Population

**Figure 1:**
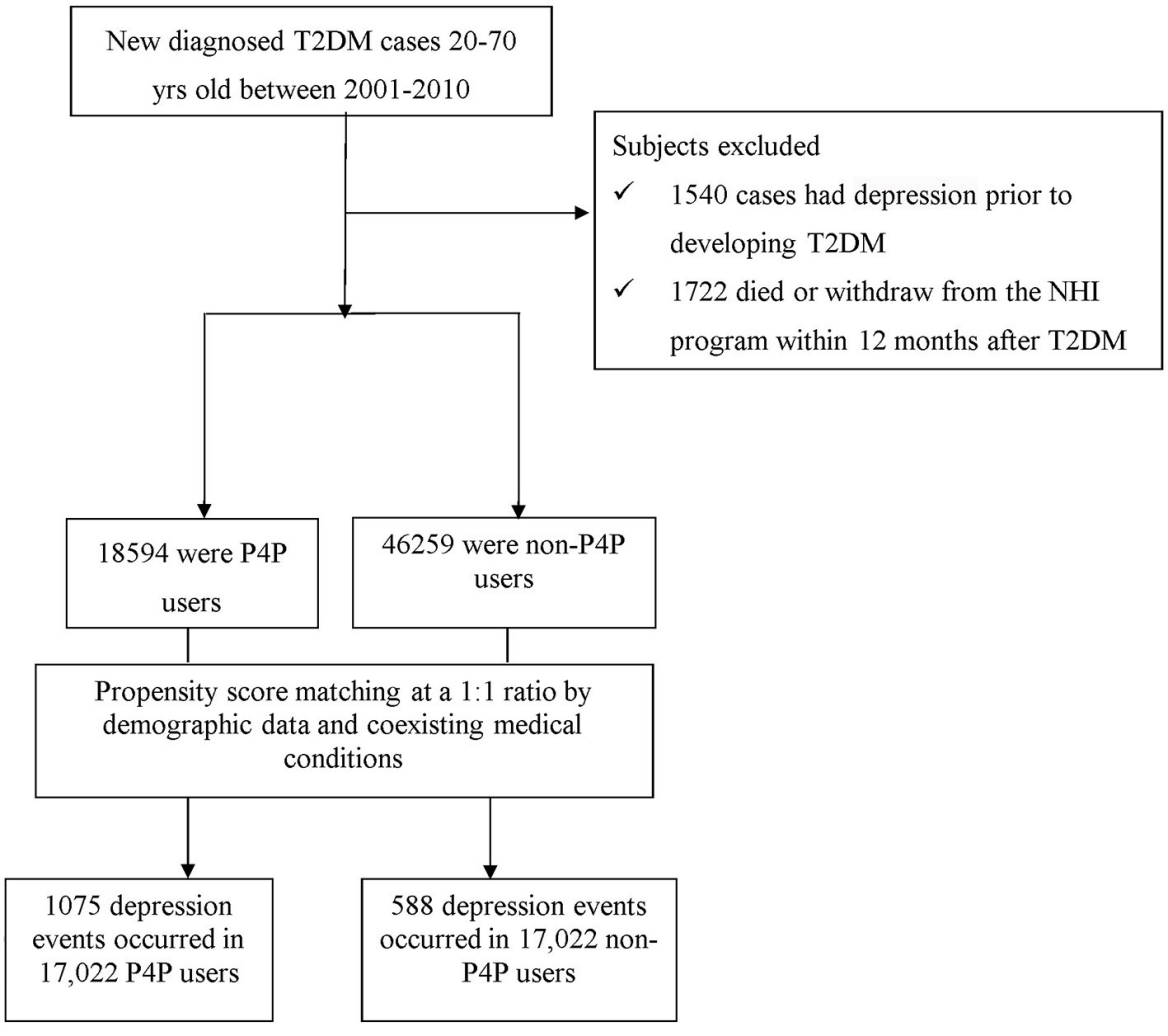
Flow chart of selection and follow up of study subjects.

Patients 20–70 years of age, newly-diagnosed with T2DM between 2001 and 2010 were identified (Figure 1). To ensure the accuracy of diagnoses, patients with T2DM were identified if they had at least three ambulatory or one inpatient claims with the International Classification of Disease, 9th Edition, Clinical Modification (ICD-9-CM) diagnosis code 250 without type 1 diabetes (ICD-9-CM code 2501). A total of 1,540 patients with T2DM were excluded because of previous diagnosis of depression (ICD-9-CM code 296.2, 296.3, 300.4, or 311). In accordance with the rationale in identifying T2DM cases, we only selected participants who had at least three outpatient visits, or at least one inpatient claim due to depression (ICD-9-CM code 296.2, 296.3, 300.4, or 311), dating from 1996, when the computerized claims data from the LHID became available, until the date of cohort entry. Those with a follow-up period <12 months were also excluded (*n* = 345). Overall, we enrolled 63,853 subjects with new-onset T2DM.

Healthcare claim data for all included patients were reviewed to determine whether the P4P model had been used following the onset of T2DM. Patients were considered as P4P users if their claim files were noted with the code of “E4” in the treatment claims after diabetes onset (13), whereas the remaining subjects were classified as non-P4P users. In Taiwan, P4P has been applied to several chronic diseases, including diabetes (13, 14) and schizophrenia (18, 19). P4P has been launched into routine clinical practice since 2001, with the aim of providing holistic care to complex or high-resource patients, particularly those with T2DM (20). This program integrated a multi-component intervention comprised of medical history, evaluation of management plan, diabetes self-management education and periodic laboratory evaluations. These evaluations contained results from blood sugar, hemoglobin A1c (HbA1c), low-density lipoprotein, triglycerides, serum creatinine, urine albumin/creatinine ratio, systolic and diastolic blood pressures, eye examination, and foot examination for initial enrollment visit. Furthermore, several of these examinations, including blood sugar, HbA1c, and systolic and diastolic blood pressures, are performed every 3 months for care visit continuity (20). Taken together, the P4P program is patient-tailored and includes assessments, planning, integration, implementation, and follow-up evaluations of treatment plans via closer collaboration between clinical specialists, such as physicians, certified diabetes educators, and registered dietitians, allowing participants to follow regular visits and healthcare providers to evaluate medication compliance and effect.

In this study, 18,594 patients were categorized as P4P users. A comparison cohort was randomly selected from the remaining insured T2DM cases without the involvement of P4P. To ensure comparability of samples, we utilized the propensity score (PS) with a 1:1 matching method to balance the characteristics between the P4P and non-P4P groups. We used multivariable logistic regression models to estimate PS-value according to all baseline characteristics (listed in Table 1) as covariates included in the model. Patients were matched by PS using one-to-one nearest neighbor matching within 0.2 caliper distance, in which, pairs of user and non-user were formed, such that matched subjects have similar values of each propensity. Afterwards, an equal number of P4P and non-P4P patients were analyzed in this study. Additionally, to consider immortal time bias that may bias the results in favor of the treatment group (21), the index date for the follow-up period for non-P4P users was the date of T2DM diagnosis, while that for P4P users was defined as the date of initiation of P4P use. The end date of the follow-up period for both groups was the earliest of the following: (a) a diagnosis of depression; (b) withdrawal from the insurance program; or (c) the date of December 31, 2012.

**Table 1:** Demographic data and selected comorbidities of study subjects.

**Variables**	**Total**	**Non-P4P users**	**P4P users**	***p***
		***N* = 17,022**	***N* = 17,022**	
**Age (year)**				0.74
≤ 50	12,279 (36.1)	6,155 (36.2)	6,124 (36.0)	
>50	21,765 (63.9)	10,867 (63.8)	10,898 (64.0)	
Mean (SD)	53.6 ± 10.1	53.6 ± 10.2	53.6 ± 10.1	0.82
**Sex**				0.88
Female	16,761 (49.2)	8,388 (49.3)	8,373 (49.2)	
Male	17,283 (50.8)	8,634 (50.7)	8,649 (50.8)	
**Monthly income**				0.36
Class 1	12,802 (37.6)	6,420 (37.7)	6,382 (37.5)	
Class 2	19,263 (56.6)	9,643 (56.7)	9,620 (56.5)	
Class 3	1,979 (5.8)	959 (5.6)	1,020 (6.0)	
**Residential area**				0.68
Urban	19,819 (58.2)	9,938 (58.4)	9,881 (58.0)	
Suburban	5,286 (15.5)	2,650 (15.6)	2,636 (15.5)	
Rural	8,939 (26.3)	4,434 (26.0)	4,505 (26.5)	
**Comorbidity**				
Hypertension	17,849 (52.4)	8,915 (52.4)	8,934 (52.5)	0.84
Obesity	621 (1.82)	315 (1.85)	306 (1.80)	0.69
Rheumatoid	350 (1.0)	239 (1.4)	211 (1.2)	0.18
Heart disease	6,311 (18.5)	3,128 (18.4)	3,183 (18.7)	0.44
Chronic kidney disease	552 (1.6)	337 (2.0)	315 (1.9)	0.38
Cancer	1,215 (3.6)	552 (3.2)	563 (3.3)	0.74
Stroke	2,324 (6.8)	1,206 (7.1)	1,218 (7.2)	0.80

### Definition of Other Covariates

Information on potential confounders included age, sex, monthly income (for estimating insurance payment), the urbanization level of enrollees' residential area, and former comorbidities. Monthly income was separated into three classes (class 1 [lowest income] to class 3 [highest income]). The urbanization level was classified into seven grades, as outlined in a previous study (22), with a higher grade indicating a more urban environment. In this study, we divided urbanization into three levels, namely, high (metropolitan cities), medium (small cities and suburban areas), and low (rural areas). Baseline comorbidities for each subject were determined by individual medical records in the year preceding cohort entry, which included hypertension (ICD-9-CM 401-405), obesity (ICD-9-CM 278.0), stroke (ICD-9-CM 430-438), heart disease (ICD-9-CM 410-429), chronic kidney disease (ICD-9-CM 585), rheumatologic disorders (ICD-9-CM 725-729), and cancer (ICD-9-CM 140-208).

### Statistical Analysis

Categorical variables are reported as frequency and/or percentage, and continuous variables are presented as mean with standard deviation (SD). In step one of the analyses, Chi-square test and *t*-test were used to examine the differences in demographic variables and comorbidities between those with and without P4P use. The incidence rate of depression was calculated as the number of cases per 1,000 person-years. Multivariate Cox proportional hazards regression was then applied to compute the hazard ratio (HR) with 95% confidence interval (CI) of depression risk in association with P4P use. To test the robustness of the relationship between P4P use and subsequent depression risk, we summed the total number of P4P use and categorized P4P usage as low, medium, or high based on the tertile distribution of P4P use. The assumption of proportional hazards was confirmed by plotting the graph of the survival function vs. the survival time and the graph of the log, log (survival), vs. the log of survival time. Statistical analyses were performed using SAS Version 9.3 software (SAS Institute Inc., Cary, NC, USA), with *p* < 0.05 considered significant.

## Results

The analysis included data from 17,022 P4P users and 17,022 non-P4P users. Analysis of the distributions of pertinent characteristics between the two groups, including age, sex, monthly income, residential area, and comorbidities, indicates that the two groups were comparable with respect to these characteristics (Table 1). The mean age for P4P users and non-P4P users were 53.6 ± 10.2 and 53.6 ± 10.1 years, respectively. The sex ratio was approximately 1:1 between males and females in both cohorts. The majority of participants had monthly income levels of Class 2 (56.6%) and tended to live in more urbanized areas (58.2%). The primary comorbidity in both cohorts was hypertension (52.4%), followed by heart disease (18.5%), and stroke (6.8%).

Among all eligible T2DM subjects, a total of 1,663 first episodes of depression occurred, 1,075 in non-P4P users and 588 in P4P users during follow-up periods of 127820.16 and 99788.27 person-years, respectively. The incidence rate of depression was found to be significantly lower in P4P users than in non-P4P users (5.89 vs. 8.41, respectively, per 1,000 person-years), with an adjusted HR of 0.73 (95% CI: 0.65–0.80) (Table 2). Subgroup analysis by low, medium, and high intensity of P4P use further indicated that T2DM patients using P4P at high intensity had a 52% lower risk of depression (95% CI: 0.41–0.58).

**Table 2:** Risk of depression for T2DM patients with and with no use of P4P program.

**Patient group**	***N***	**Events**	**Person-years**	**Incidence**	Adjusted HR^*^ (95% CI)
Non-P4P users	17,022	1,075	127820.16	8.41	1
P4P users	17,022	588	99788.27	5.89	0.73 (0.64–0.83)
Low intensity	8,497	287	42338.85	6.78	0.85 (0.75–0.98)
Medium intensity	4,425	163	24126.49	6.76	0.84 (0.71–0.97)
High intensity	4,100	138	33322.93	4.14	0.48 (0.41–0.58)

* *Model adjusted for sex, age, residential area, monthly income, and comorbidities*.

We performed an additional stratified analysis by age and sex to determine the effect of P4P on the risk of depression. In general, the use of P4P was associated with a lower risk of depression, regardless of subject's sex or age. Multivariable stratified analysis showed that the likelihood of decreased depression was greater in males than females (adjusted HR: 0.67; 95% CI: 0.58–0.81) (Table 3).

**Table 3:** Incidence and depression risk for T2DM patients with and with no P4P use stratified by sex and age.

**Variables**	**Non-P4P users**			**P4P users**			**Adjusted HR (95% CI)**
	**Patients**	**Person-years**	**Incidence**	**Case**	**Person-years**	**Incidence**	
**Sex**							
Female	652	62988.59	10.35	372	49585.20	7.50	0.75^Y^ (0.65–0.84)
Male	423	64831.57	6.52	216	50203.07	4.30	0.67^Y^ (0.58–0.81)
**Age**							
≤ 50	347	44602.88	7.78	192	34874.25	5.51	0.74^*^ (0.62–0.89)
>50	728	83217.28	8.75	396	64914.02	6.10	0.73^*^ (0.63–0.87)

Y * Model adjusted for age, residential area, monthly income, and comorbidities*.

* *Model adjusted for sex, residential area, monthly income, and comorbidities*.

## Discussion

Findings of this retrospective cohort study indicated that depression risk was lower for T2DM patients who received P4P than for those who did not. Multivariable analysis showed that P4P use was associated with a 73% lower risk of depression among T2DM subjects. High-intensity use of P4P had markedly greater benefits, with a 52% lower risk of depression. As a dose-response relationship is considered strong evidence for a causal relation between exposure level and outcome, this finding suggests that P4P use is indeed successful in decreasing the likelihood of depression. While studies on this issue are scarce, this positive therapeutic effect is consistent with earlier reports and adds to a growing body of evidence regarding the clinical effectiveness of P4P (12–14, 23).

We suggest several potential reasons for the beneficial effect of P4P on depression risk in this study. First, a unique difference between the P4P program and conventional treatment is that the former utilized a highly interactive approach to more actively engage T2DM patients with educational and therapeutic information, throughout the post-implementation period (10, 20). This approach might be beneficial in instituting individually-tailored, need-based strategies for these patients to increase their acceptance of T2DM and ultimately improve their psychological adjustment. Of the studies conducted thus far, some have shown that interventions such as structured self-monitoring of blood glucose, consecutive group-based counseling, and an education program were related to lower risks of depression following T2DM diagnosis (7, 24). Second, the continuity of care embedded in P4P is reported to enable diabetic patients to maintain good metabolic control, especially of their HbA1c levels (25). For diabetic patients, higher HbA1c is thought to correlate with a higher risk of developing depression (26, 27). Previous studies have shown that patients with poorly controlled diabetes commonly had higher levels of the pro-inflammatory cytokines tumor necrosis factor-α (TNF-α), interleukin 1 (IL-1), IL-6, and HbA1c-values (28, 29). These mediators may play important roles in the pathogenesis of neuropsychiatric symptoms, especially depression (30).

Age-and sex-specific analyses showed that P4P provides greater benefits for male subjects. Two reasons may account for this phenomenon. First, women have been shown to be more health-conscious than men and thus may be more likely to pursue treatment at the earliest notice of medical irregularity (31), thereby lessening or moderating the preventive effect of the P4P program in influencing depression onset. In addition, females may benefit from inherent estrogen, which is known to suppress production of the inflammatory cytokines IL-1, IL-6, and TNF-α (32), which may affect the effect of P4P among them.

As previously described in the Methods section, the database used in this study has several strengths, including its representativeness of the entire Taiwanese population and the large sample size that ensured reliable findings. In addition, this study is the first to investigate the relationship between P4P use and depression risk in patients with T2DM by using a longitudinal cohort study design, thus allowing us to robustly examine the relationship between high intensity P4P use and the risk of depression among such patients. Nonetheless, several limitations of this study merit attention. First, the use of secondary health care databases might pose the risk of errors in the coding process. To diminish this potential risk, the disease of interest in this study, namely T2DM, as well as the manifestations of depression and comorbidities, were identified if it ever occurred at a minimum of three outpatient visits, or if, at least, one inpatient admission during the follow-up period was recorded. Additionally, the NHI of Taiwan randomly reviews the charts and audits medical charges to verify the accuracy of claims (15). Moreover, because the coding approach and data availability were similar for the two cohorts, it could be argued that this similarity would only tend to underestimate, rather than overestimate, the magnitude of exposure—disease association. Second, the LHID lacks information on social network relationships, family history, personality attributes, laboratory data, and body mass index. Thus, we used several available comorbidities as surrogates to address these unmeasured confounders. For example, obesity and hypertension were used to substitute for the influence of body mass index and physical inactivity (33). Future research, however, addressing these factors is needed to expand on these preliminary findings. Third, although our study revealed a substantial benefit resulting from the use of P4P for T2DM patients, it must be recognized that these participants were not randomly categorized into users and non-users. Data derived from a retrospective cohort study are generally of lower statistical quality than those derived from randomized trials because of potential biases. Therefore, caution should be exercised when interpreting the findings. A large-scale randomized controlled trial is, therefore, recommended to better determine the efficacious influence of P4P on other psychiatric disorders among diabetic patients.

## Conclusion

This large-scale cohort study is the first to clarify whether adding the P4P into the routine care process could lessen the subsequent depression risk among T2DM patients. Our results show that the implementation of P4P indeed decreases the subsequent risk of depression by 27%. Notably, we observe an additional positive effect associated with high-intensity P4P for such patients. The current study provides further evidence that the integration of P4P programs into routine disease management may be beneficial for achieving treatment goals and reducing costs for patients with chronic diseases.

## Data Availability Statement

The data supporting the conclusion of this study are available from the authors, but the raw data (NHIRD) need to be obtained from the National Health Research Institute of Taiwan through an application process upon approval.

## Ethics Statement

The studies involving human participants were reviewed and approved by Institutional Review Board and Ethics Committee of Buddhist Dalin Tzu Chi Hospital (No. B10004021-3). Written informed consent for participation was not required for this study in accordance with the national legislation and the institutional requirements.

## Author Contributions

W-CL, HL, H-JH, and T-YT were involved in the study design and drafted the manuscript. W-CL, HL, H-RG, and T-YT contributed to the data analysis and revised manuscript and were responsible for the study conception, design, data analysis, and drafting. W-CL, H-JH, M-CL, and T-YT contributed to the interpretation of data and provided comments on the final draft of the manuscript. M-CL and T-YT provided administrative support and comments on the manuscript drafts. All authors gave final approval of the version to be published, and agree to be accountable for all aspects of the work.

## Conflict of Interest

The authors declare that the research was conducted in the absence of any commercial or financial relationships that could be construed as a potential conflict of interest.

## Publisher's Note

All claims expressed in this article are solely those of the authors and do not necessarily represent those of their affiliated organizations, or those of the publisher, the editors and the reviewers. Any product that may be evaluated in this article, or claim that may be made by its manufacturer, is not guaranteed or endorsed by the publisher.
